# Unipolar and Bipolar Depression : Which Differences?

**DOI:** 10.1192/j.eurpsy.2024.909

**Published:** 2024-08-27

**Authors:** M. Turki, A. Labyadh, W. Abid, N. Halouani, S. Ellouz, J. Aloulou

**Affiliations:** ^1^Psychiatry B department; ^2^Psychiatry C department, Hedi Cheker Hospital, Sfax, Tunisia

## Abstract

**Introduction:**

Depression is a common mental disorder whose management remains delicate, given the trans-nosographic nature of this syndrome. Two common types of depression are bipolar and unipolar depression. Although they share many similar symptoms, several differences between the two pathologies are suggested in prior studies.

**Objectives:**

We aimed to compare the disease characteristics and evolution of unipolar and bipolar depressed patients.

**Methods:**

We conducted a retrospective descriptive and analytical study among medical records of 167 patients hospitalized for a depressive episode (DE) at the Psychiatry “B” Department, Hedi Chaker University Hospital (Sfax, Tunisia), during the period between 2015 and 2017. Patients were divided into two groups according to DSM-5 criteria: those with bipolar disorder I or II (bipolar depression) versus those with major depressive disorder (unipolar depression).

**Results:**

The mean age of our patients was 37.6 years, with a female predominance (sex-ration F/M =1.7). The age of onset of the disease was earlier in bipolar depressed patients (29.36 versus 31.89), without a significant relationship. Family psychiatric history was significantly more prevalent in bipolar disorder patients (73.5% versus 37.3%; p<0.001). Bipolar patients are more likely to be unemployed (65.3% versus 50.8%), but without a significant relationship.

Bipolar patients were more likely to be hospitalized for suicide attempts (44.9% versus 35.6%; p=0.2).

**Conclusions:**

Distinguishing between major depressive disorder and bipolar disorder is important because there are differences in the optimal management of these conditions.

**Disclosure of Interest:**

None Declared

